# Vitamin D in Depression: A Potential Bioactive Agent to Reduce Suicide and Suicide Attempt Risk

**DOI:** 10.3390/nu15071765

**Published:** 2023-04-04

**Authors:** María Montserrat Somoza-Moncada, Francisco Javier Turrubiates-Hernández, José Francisco Muñoz-Valle, Jesús Alberto Gutiérrez-Brito, Saúl Alberto Díaz-Pérez, Adriana Aguayo-Arelis, Jorge Hernández-Bello

**Affiliations:** 1 Instituto de Investigación en Ciencias Biomédicas, Centro Universitario de Ciencias de la Salud (CUCS), Universidad de Guadalajara (UdeG), Guadalajara 44340, Jalisco, Mexico; maria.somoza2711@alumnos.udg.mx (M.M.S.-M.); francisco.turrubiates3337@alumnos.udg.mx (F.J.T.-H.); drjosefranciscomv@cucs.udg.mx (J.F.M.-V.); jesus.gutierrez3225@alumnos.udg.mx (J.A.G.-B.); saul.diaz2330@alumnos.udg.mx (S.A.D.-P.); 2 Doctorado en Ciencias de la Nutrición Traslacional, Centro Universitario de Ciencias de la Salud (CUCS), Universidad de Guadalajara (UdeG), Guadalajara 44340, Jalisco, Mexico; 3 Departamento de Neurociencias, Centro Universitario de Ciencias de la Salud (CUCS), Universidad de Guadalajara (UdeG), Guadalajara 44340, Jalisco, Mexico; adriana.aguayo@academicos.udg.mx

**Keywords:** vitamin D deficiency, calcitriol, major depressive disorder, self-injurious behavior, suicidal ideation, neuropsychiatry, neuroinflammation, serotonin, cytokines, kynurenine

## Abstract

Suicide is one of the leading causes of death worldwide. According to the World Health Organization (WHO), every year, more than 700 thousand people die from this cause. Therefore, suicide is a public health issue. The complex interaction between different factors causes suicide; however, depression is one of the most frequent factors in people who have attempted suicide. Several studies have reported that vitamin D deficiency may be a relevant risk factor for depression, and vitamin D supplementation has shown promising effects in the adjunctive treatment of this mood disorder. Among the beneficial mechanisms of vitamin D, it has been proposed that it may enhance serotonin synthesis and modulate proinflammatory cytokines since low serotonin levels and systemic inflammation have been associated with depression and suicide. The present narrative review shows the potential pathogenic role of vitamin D deficiency in depression and suicide and the potential benefits of vitamin D supplementation to reduce their risk.

## 1. Introduction

Suicide is a death caused by self-injurious behavior with evidence of an intentional desire to die [1]. In contrast, non-fatal suicidal thoughts and behaviors are classified as follows: (a) suicidal ideation (passive), which refers to a series of desires and contemplations of carrying out a suicidal act; (b) suicidal plan (active), which is the planning of methods with which one intends to end one’s own life; and (c) suicide attempt, which refers to self-injurious behavior with intent to die that may not result in physical injury [1,2].

According to information from the WHO, more than 700,000 suicides occur yearly. In 2019, suicide represented the fourth leading cause of death worldwide in individuals aged 15 to 29 years. Seventy-seven percent of these suicides occurred in low- and middle-income countries; however, suicide has a global impact that affects even high-income populations [3]. The COVID-19 pandemic exacerbated the risk factors associated with suicide or suicidal behavior. The mental health impact of social distancing, quarantines, and financial crises due to loss of employment was identified as a significant risk factor associated with this exacerbation [4]. Therefore, the WHO recognizes these problems as a priority public health issue [3].

Suicide can be caused by a dynamic and complex interaction between several factors [5], such as mental disorders, genetic risk factors, availability of firearms, and bad life experiences [6]. However, mental disorders account for the vast majority (between 60% and 98%) of suicides and suicide attempts [7]. According to a recent meta-analysis, a mental disorder increases the risk of suicide almost ten times and the risk of suicide attempt almost three times [8].

Depression is a mental disorder that causes persistent sadness and loss of interest. Among mental disorders, criteria for depression have been identified in up to 65% of suicide cases [5,9]. Likewise, a positive association between depression and the risk of a suicide attempt has been reported (OR = 1.54 (95%CI: 1.21–1.96), *p* < 0.001) [8].

Vitamin D is a micronutrient identified as a hormone because its active metabolite —calcitriol—exerts endocrine, paracrine, and autocrine effects [10]. The classical function of vitamin D is the regulation of calcium and phosphorus serum levels in a healthy physiological range [11]; however, calcitriol also exerts functions involved in the cardiovascular system, neurodevelopment, and immune response [12,13,14,15,16].

According to previous research, vitamin D deficiency may be a risk factor for depression [17,18] and suicide attempts [19,20]. This could be explained because vitamin D deficiency can alter the availability of some cholinergic, dopaminergic, and noradrenergic neurotransmitters, which have been implicated in depression [21]. In addition, some studies have shown that vitamin D supplementation may improve the clinical depressive state [22,23]. Vitamin D also can modulate proinflammatory cytokines (e.g., IL-6 and TNF-α), which are essential in systemic inflammation associated with depression and suicide [24,25].

This narrative review addresses an overview of the pathogenic role of vitamin D deficiency in depression, suicide, and suicide attempt.

## 2. Material and Methods

A search was conducted using the PubMed/MEDLINE electronic database without restrictions on publication dates. The included studies were mainly original articles, narrative reviews, systematic reviews, and meta-analyses, and the language was restricted to English. Titles and abstracts were considered for full-text review. The search strategy was performed based on the following keywords: “depression”, “mood disorder”, “suicide attempt” “suicide”, “vitamin D”, “vitamin D intake”, “dietary vitamin D”, “vitamin D deficiency”, “calcidiol”, “calcitriol”, “vitamin D receptor”, “vitamin D supplementation”, “neurotransmitters”, “serotonin”, “inflammation”, and “neuroinflammation”. However, complementary bibliography beyond the established keywords was also considered. Further screening of studies was conducted by reading the full text of the papers according to the aim of this review. Studies with a small sample size (<20) were excluded because of low statistical power.

## 3. Vitamin D

### 3.1. Vitamin D Sources and References Levels

Vitamin D is the generic term for ergocalciferol (vitamin D_2_) and cholecalciferol (vitamin D_3_) which can be found mainly in plant and animal sources, respectively. However, it is estimated that most vitamin D_3_ in humans could be synthesized in the epidermis from 7-dehydrocholesterol (7-DHC), a precursor of cholesterol [26]. Ultraviolet-B (UVB) rays (290–315 nm) from the sun penetrate the epidermis, where 7-DHC absorbs the energy to be cleaved and form previtamin D_3_. Subsequently, through a thermosensitive process, previtamin D_3_ is isomerized to form vitamin D_3_ [27]. Nevertheless, despite the relevance of UVB rays as a source of vitamin D, it should be noted that several factors can reduce its syntheses in the epidermis, such as sunscreen use, skin pigmentation, aging, geographical latitude, season, and time of day [28].

Calcidiol (25[OH]D) is the reference biomarker for the determination of clinical vitamin D status due to its half-life of up to three weeks in circulation [12]. The Endocrine Society (USA) and the European Society of Endocrinology specify vitamin D sufficiency when there is a calcidiol concentration >30 ng/mL (75 nmol/L), insufficiency of 20 to 30 ng/mL (50 to 75 nmol/L), and deficiency <20 ng/mL (50 nmol/L) [29]. Maintaining a calcidiol concentration between 40 and 60 ng/mL (100–150 nmol/L) is suggested to obtain the best health benefit from vitamin D [30,31,32].

### 3.2. Vitamin D Metabolism

**Figure 1:**
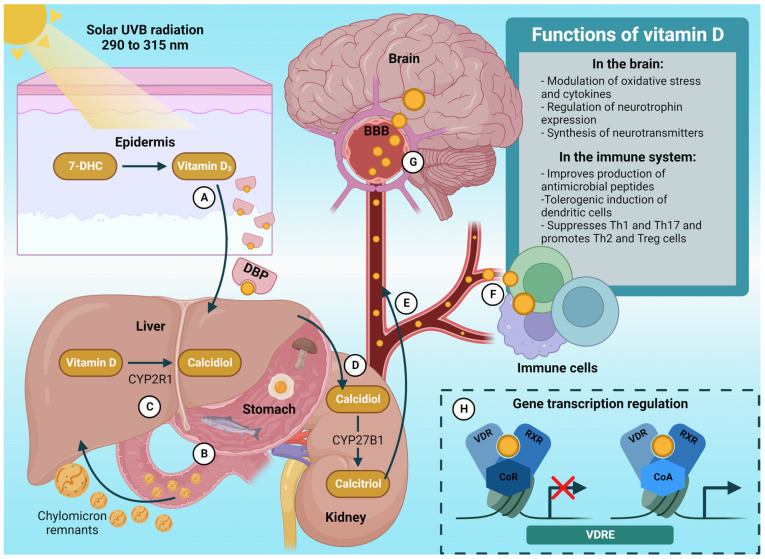
Metabolism, signaling, and functions of vitamin D. (**A**) Vitamin D_3_ synthesized in the skin binds to DBP for its transport into circulation. (**B**) Dietary vitamin D (ergocalciferol or cholecalciferol) is incorporated into chylomicrons that promote its absorption and transport to the liver. (**C**) Vitamin D_2_ and D_3_ are metabolized in the liver by CYP2R1 for the formation of calcidiol. (**D**) In the kidneys, calcidiol is metabolized to calcitriol by CYP27B1. (**E**) Calcidiol is mobilized in the circulation for extrarenal metabolism, while calcitriol is mobilized to exert endocrine functions. (**F**) Calcidiol can be metabolized in immune cells (macrophages and lymphocytes), which in turn express VDRs to exert paracrine and autocrine functions. (**G**) Calcidiol can cross the blood-brain barrier (BBB); moreover, calcitriol produced in the brain exerts several regulatory functions (based mainly on experimental studies). (**H**) Calcitriol interacts with its nuclear receptors to form a complex (VDR–RXR) on VDREs. This complex in collaboration with nuclear corepressors or coactivators, regulates the transcription of target genes. UVB, ultraviolet-B; 7-DHC, 7-dehydrocholesterol; Vitamin D_2_, ergocalciferol; Vitamin D_3_, cholecalciferol; DBP, vitamin D binding protein; CYP2R1, cytochrome P450 Family 2 Subfamily R Member 1 (25-hydroxylase); CYP27B1, cytochrome P450 Family 27 Subfamily B Member 1 (25-hydroxyvitamin D-1 alpha hydroxylase); VDR, vitamin D receptor; RXR, retinoid X receptor; CoR, nuclear corepressors; CoA, nuclear coactivators; VDRE, vitamin D response element; Th, T helper cells; Tregs, T regulatory cells.

Vitamin D synthesized in the skin is attracted to the dermal capillary bed by the vitamin D binding protein (DBP), which transports it to the liver for its metabolism [11,33] (Figure 1A). Likewise, vitamin D from the diet is incorporated into chylomicrons for release into the lymphatic system. Subsequently, chylomicrons remnant particles in the circulation deliver dietary vitamin D to the liver [11,34] (Figure 1B). Once in the liver, CYP2R1 hydroxylates both exogenous and endogenous vitamin D for the production of calcidiol, the primary metabolite of vitamin D in the circulation [28,35] (Figure 1C). After that, CYP27B1 hydroxylates calcidiol in the kidneys to form 1,25-hydroxyvitamin D (1,25[OH]_2_D), also termed calcitriol. The latter is responsible for exerting the biological functions of this vitamin [11,28,36] (Figure 1D,E).

Calcitriol is the ligand for vitamin D receptor (VDR), a member of the nuclear receptor family of transcription factors which activates or represses the expression of nearly 1000 genes in many cell types, including immune cells, given the wide distribution of VDR [37] (Figure 1F,H). Upon interaction with calcitriol, the VDR is activated and forms a heterodimer with the retinoid X receptor (RXR). This complex (VDR–RXR) binds to vitamin D response elements (VDREs) in DNA, where corepressors or coactivators with histone-modifying activity are assembled, allowing the regulation of transcription of genes [38] (Figure 1H).

Calcidiol and calcitriol are present in the brain [39,40]. In brain cells, changes in vitamin D status have been associated with impaired cytokine regulation and affect cell differentiation, neurotrophin expression, intracellular calcium signaling, neurotransmitter release, anti-oxidant activity, anti-inflammatory actions, stress responsivity, and the expression of genes/proteins essential to neuron physiology [41] (Figure 1G).

### 3.3. Vitamin D Is a Bioactive Agent in the Brain

Although VDR was initially identified in tissues related to calcium and phosphorus homeostasis (intestine, bones, kidneys, and parathyroid gland), it is now recognized that VDR is also present in brain cells (astrocytes, microglia, and neurons) and immune system cells (T cells, B cells, and macrophages). Therefore, vitamin D can perform several brain immunoregulatory functions [42,43,44,45]. Some authors have demonstrated possible cognitive-enhancing effects of vitamin D, which may reflect a direct action in the brain rather than a result of secondary systemic effects. Indeed, in experimental rodent models, vitamin D has direct neuroprotective actions and can reduce some biomarkers of brain aging, i.e., optimal levels of vitamin D stabilize myelin structure and enhance synaptic vesicle recycling and transcription factors facilitating cognitive processes [46,47,48]. Moreover, CYP27B1 and VDR have been prominently reported in the hypothalamus and the large (presumably dopaminergic) neurons within the substantia nigra. Therefore, vitamin D could has similar functions to other neurosteroids and may have autocrine/paracrine properties in the human brain [49]. Additionally, preclinical studies indicating vitamin D deficiency in early life affect neuronal differentiation, axonal connectivity, dopamine ontogeny, and brain structure and function. These bioactive mechanisms offer an intriguing possibility of the epidemiological associations between vitamin D deficiencies and psychiatric disorders such as depression [21,50,51,52].

## 4. Depression: A Significant Risk Factor for Suicide and Suicide Attempt

Suicide and suicidal behavior comprise the sixth and ninth leading causes of global disease burden among men and women 15 to 44 years, respectively [53]. Factors leading to suicide may be divided into predisposing and precipitating stressors [54]. Some predisposing factors identified for suicide include psychiatric disorders, previous suicide attempts, substance abuse, hopelessness, and a family history of suicidal behavior [55,56]. It has been reported that up to 15% of patients with recurrent depressive disorder commit suicide [57].

The association of suicide with depression, particularly in major depressive disorder (MDD), can be explained by the synergic role of genetics, endogenous and exogenous stressors, epigenetics, the hypothalamic–pituitary–adrenal stress-response system, the involvement of the monoaminergic neurotransmitter systems, neuro-immunological biomarkers, the brain-derived neurotrophic factor, and other neuromodulators [58].

### 4.1. Neurological Mechanisms Associated with Depression and Suicide

Serotonin has been highlighted as a principal neurotransmitter altered in depression; therefore, selective serotonin reuptake inhibitors (SSRI) tend to be the first-line treatment. The monoamine hypothesis of depression [59] suggests that the deficit of monoamines (serotonin, norepinephrine, and dopamine) in the brain is the basis of the pathogenesis of depressive disorder [60,61].

Serotonin is a tryptophan-derived neurotransmitter that can be synthesized in the brain by tryptophan hydroxylase 2 (TPH2) [23,62]. There are two main pathways in tryptophan metabolization: one is the kynurenine (KYN) pathway, initiated by the enzyme indoleamine 2,3-dioxygenase (IDO), and the other is the serotonin pathway [63].

Dysregulation of the KYN pathway was reported in suicidal patients for the first time by Sublette et al. (2011), who associated higher plasma KYN concentrations with a history of suicide attempts [64]. Moreover, increased quinolinic acid (QUIN) levels (a metabolite in the KYN pathway) have been associated with behavioral symptoms of depression and suicidality in other studies afterward [65,66,67].

The monoamine hypothesis is also supported by data showing that monoamine oxidase inhibitors are effective antidepressants and appear to work by increasing serotonergic and noradrenergic signaling [68]. However, these drugs can fail to produce a rapid and sustained antidepressant response in a substantial proportion (until 2/3) of depressed patients; therefore, the neurobiological mechanisms of depression cannot be explained solely as a consequence of these mechanisms [60,69].

Another system involved in the neuropathogenesis of depression is the hypothalamic–pituitary–adrenal (HPA) axis, one of the major endocrine systems responsible for maintaining homeostasis when the individual is challenged or stressed [70]. Alterations in the HPA axis are common in depression and are associated with suicide risk, regardless of the presence or absence of depression [71]. Furthermore, noradrenergic overactivity due to an overactivity of the HPA axis has been associated with higher suicide risk [71,72].

### 4.2. Inflammatory Mechanisms Associated with Depression and Suicide

The presence of inflammation markers in depression has been reported in different studies; especially, overproduction of proinflammatory cytokines has been reported in several neuropsychiatric conditions, including MDD [73,74,75,76]. Patients with MDD and suicidal attempts have been found to have increased levels of IL-6, TNF-α, and C-reactive protein (CRP), with decreased levels of anti-inflammatory cytokines such as IL-10. Moreover, patients with arthritis—a chronic inflammatory disease—have significantly higher odds of committing suicide attempts in comparison to controls, and this trend is still significant after adjusting for well-known confounding variables such as adverse substance abuse, history of depression and anxiety, and current pain level [77].

Additionally, it has been reported that 1/3 of patients receiving interferon treatment develop depressive-like symptoms during therapy [78]. Moreover, individuals who receive injections of lipopolysaccharide (LPS), which induces a systemic inflammatory response, experience depressive symptoms [79].

A post-mortem study provided evidence of increased inflammation in the brains of suicide victims associated with depression [80]. Moreover, evidence for aberrant cytokine levels in blood, cerebrospinal fluid, and post-mortem brain samples of patients with suicidality was reported in a meta-analysis study. Especially, IL-1β and IL-6 were most robustly associated with suicidality, and the authors suggested that these cytokines may help distinguish suicidal from non-suicidal patients [81].

In a recent literature review, Lena Brundin et al. summarized a great deal of evidence that implicates dysregulation of the immune system in the pathophysiology of depression and suicidality. They include various inflammatory conditions, such as traumatic brain injury, vitamin deficiency, autoimmune disorders, and infections, which, through raised levels of inflammatory mediators, can cause hyperactivation of the HPA axis and alterations in monoamine metabolism in the patients, causing changes in emotion and behavior, which could ultimately lead to suicide in vulnerable individuals [76].

## 5. Vitamin D, Depression, and Suicide: Interrelated Evidence and Mechanisms

### 5.1. Sun and Depression

Seasonal affective disorder (SAD) is characterized by hopelessness, fatigue, and depression [82,83]. The recurrence of this condition is almost annual since the symptoms appear during seasonal changes, particularly in autumn and winter [84]. Interestingly, the symptoms disappear once the seasonal period is over; therefore, SAD is classified as a depression related to climate and seasonal weather changes [83].

It has been hypothesized that SAD may be associated with a lack of sunlight due to its incidence during certain year seasons [85]. In particular, it is believed that sunlight deficiency may alter the synthesis of neurotransmitters related to regulating circadian rhythm and mood (e.g., melatonin and serotonin) [82,83]. Since SAD is a cognitive disorder, its approach is mainly focused on cognitive–behavioral therapy, which is usually accompanied by antidepressants and light therapy. Moreover, vitamin D supplementation has been proposed as an adjuvant treatment because of the potential relevance of serotonin in the pathophysiology of SAD [83]. The latter is based on the critical influence of this vitamin on the synthesis and concentration of serotonin [62,86].

### 5.2. Vitamin D Levels, Depression, and Suicide

Different sources of chronic inflammation, such as stress, trauma, short sleeping hours, and sedentary habits, are related to depressive disorder [87]. Therefore, inflammation, depression, and suicide are associated, but the underlying mechanism is still unknown; despite this, vitamin D has been proposed as a common element in these conditions [76].

Patients with depressive symptoms had a lower calcidiol concentration compared to those without depression, even after adjustment with other variables such as age, gender, and body mass index [88]. Low calcidiol levels have also been associated with specific predisposing factors for suicide, such as exacerbation of depression and other psychiatric disorders [89]. Table 1 and Table 2 summarize some experimental and observational studies retrieved from this scoping that have attempted to trace the relationship between vitamin D, depression, and suicide. These studies present evidence that broadens the discussion associating vitamin D with depressive symptoms and suicidal behavior. Nevertheless, due to the heterogeneity of risk factors associated with depression and the disparity of association with suicidal behavior, the authors of the discussed reports encourage additional studies to elucidate this further.

Most of the literature collected for this discussion comes from cross-sectional studies [18,24,90,91,92], which do not allow for establishing causality. Moreover, the multifactorial nature of depression and suicidal behavior per se complicates exploring isolated associations they might have with vitamin D; therefore, it is fundamental that further studies control in more rigorous wat the most common confounding variables. Authors have kept this in mind and recorded somatic illnesses, medications, and sampling seasons of vitamin D; however, failure to control for some comorbidities [91], ethnicity, and smoking habits [90] are scant.

Perhaps the most overlooked confounding variable was exposure to UVB, but hypovitaminosis D is more related to urban residency and air pollution, regardless of a high sun exposure index [93]. One study measured urbanization and found it associated with depression in elderly individuals [94].

Vitamin D deficit was associated with inflammatory markers in depressed and suicidal individuals [24,90], depressive symptoms and severity [18,94], suicidal risk [91], and affect [95]. On the contrary, one study found no association [92]; some limitations could explain this discrepancy with the summarized studies. For example, the discrepant study did not measure depression and suicidal behavior through a validated instrument, and the cases differed significantly from those without mood alterations.

On the other hand, randomized controlled trials only assessed short-term supplementation, except for one, which carried out a one-year vitamin D supplementation [96]. This long-term study cannot wholly extrapolate to the rest randomized trials because the evaluated population consists of overweight patients.

One study of the short Vitamin D supplementations increased the positive affect but did not measure vitamin D and used an instrument with a floor effect, which hindered the interpretation of the negative affect [95]. Additionally, other short-term supplementations with vitamin D showed that eight-week supplementation with 50,000 IU/2 weeks of vitamin D elevated the calcidiol concentration of subjects with mild to moderate depression and significantly improved their depression severity [23].

It is undeniable that the evidence is still inconclusive. However, as anti-depressive medication has a significant clinical impact on patients at the upper end of the very severely depressed category [97] is valid to explore the value of vitamin D supplementation for certain groups of individuals, such as people who are overweight, people living in urban areas, older adults, or patients with elevated immune markers.

Considering the reported associations of vitamin D with depression and suicide, more rigorous studies, such as clinical trials, are needed to determine vitamin D supplementation’s role in the adjunctive treatment of depressive disorders and suicidal behavior.

**Table 1:** Observational studies associating vitamin D, depression, and suicidal behavior.

Author, Year	Country; *N*; Age	Objective(s)	Vitamin D Measure	Depression Measure	Suicidal Behavior	Additional Outcomes	Results/Conclusion
Grudet et al., 2014 [90]	Sweden; 59 suicide attempters (25 men and 34 women): non-suicidal depressed patients (*n* = 17) and healthy controls (*n* = 14); 18–73 years old	Asses the association between suicide attempt, vitamin D, and inflammatory changes	Calcidiol was measured in plasma using liquid chromatography–mass spectrometry (LC–MS)	Diagnosis according to the Diagnostic and Statistical Manual of Mental Disorders IV (DSM-IV) as schizoaffective disorder (*n* = 2), Psychotic Disorder (*n* = 1), Major Depressive Disorder (*n* = 10), Bipolar I Disorder (*n* = 3), Bipolar II Disorder (*n* = 12), Anxiety disorder (*n* = 4), Generalized Anxiety Disorder (*n* = 1), Dysthymic Disorder (*n* = 4), Alcohol Dependence (*n* = 6), Substance Dependence (*n* = 2), Adjustment Disorder (*n* = 7), Adjustment Disorder with Depressed Mood (*n* = 3), and Depressive Disorder	Suicide attempt	Vitamin D’s association with plasma IL-1β, IL-6, and TNF-α	In comparison to depressed non-suicidal patients and healthy controls, patients with suicide attempts had significantly lower mean vitamin D levels. Clinically, 58% of the suicide attempters had vitamin D deficiency. Increased levels of IL-6 and IL-1β in the blood were associated with low vitamin D. A deficiency in vitamin D was found in suicide attempters. The results suggest this deficiency might contribute to higher proinflammatory cytokines previously found in suicidal individuals.
Il Park et al., 2016 [92]	Republic of Korea; 15,695 subjects; 20 years and older	Explore the relationship among depressive symptoms, suicidal ideation, and vitamin D in a representative sample of the general population	Serum calcidiol levels were measured by radioimmunoassay (DiaSorin, Stillwater, MN, USA) using a gamma counter (1470 Wizard; Perkin Elmer)	Depressive symptoms (“yes” vs. “no”) were evaluated by asking, “Have you felt so sad or hopeless for at least two consecutive weeks during the past year that you had difficulty performing your usual activities?”	Self-reported information about suicidal ideation (‘‘yes’’ vs. ‘‘no’’) was evaluated by asking, “Did you ever feel like committing. suicide during the past year?”	Sociodemographic and health-related factors	No significant differences in serum 25-hydroxyvitamin D concentrations were found among depressive symptoms and suicidal ideation. Vitamin D, depressive symptoms, and suicidal ideation were not significantly associated. Further studies could help elucidate further the association or lack of association between vitamin D, depressive symptoms, and suicidal ideation.
Grudet et al., 2020 [24]	USA; 48 un-medicated major depressive disorder (MDD) subjects and 54 healthy controls; 39.3 ± 14.9 years old	Asses the association between suicide ideation, vitamin D, and inflammatory markers in patients with MDD	Analyses of calcidiol were conducted by liquid chromatography–mass spectrometry, model Sciex API 4000 LC/MS/MS	The Structured Clinical Interview for DSM-IV-TR Axis I Disorders (SCID); clinical interview with a board-certified psychiatrist. Depression severity: 17-item version of the Hamilton Depression Rating Scale (HDRS).	MDD subjects were categorized as “non-Suicidal Ideation group” or “Suicidal Ideation group” based on their HDRS suicidality item score. Subjects indicating a suicide attempt or current suicidal intent within the past week were excluded from the study.	Inflammatory markers IL-6 and TNF-α, neutrophil-to-lymphocyte ratio (NLR), and white blood cell count (WBC)	Patients with MDD with and without suicidal ideation (SI) did not display significant differences in calcidiol levels when compared between them and controls. All the measured inflammatory markers were negatively correlated with calcidiol; these correlations were more significant in MDD subjects, especially in the SI group. Even though calcidiol levels did not discriminate MDD with or without SI or vs. controls, indicators of immune activation in MDD were associated with lower calcidiol, particularly in cases with SI.
Grudet et al., 2022 [18]	Sweden; 202 patients and 41 healthy Controls; 18–77 years old	(a) Compare calcidiol levels between clinically depressed individuals with insufficient treatment response and healthy controls; (b) assess the association between different affective disorder diagnoses, grade of suicidal ideation, and calcidiol levels	Analyses of calcidiol were conducted by liquid chromatography–mass spectrometry, model Sciex API 4000 (LC/MS/MS)	Diagnosis made according to the DSM-IV-TR; International Neuropsychiatric Interview (MINI) 6.0; SCID-II. Current psychiatric symptoms were assessed using the Comprehensive Psychopathological Rating Scale (CPRS), and the Montgomery-Åsberg Depression Rating Scale (MADRS) was extracted. Patients were divided into four groups based on their diagnosis: major depressive disorder (MDD) single episode (*n* = 17), MDD recurrent episode (*n* = 101), chronic MDD (*n* = 59), or dysthymia (*n* = 18).	Suicidal ideation (SI) was assessed by the Suicide Assessment Scale (SUAS-S). Subjects are divided into high-grade suicidal ideation and low-grade suicidal ideation.		Patients with depression that had not remitted with previous and ongoing treatments at the moment of the study had significantly lower levels of calcidiol than healthy controls. Only 5% of the controls were calcidiol deficient (<50 nmol/L), while 30% of the depressed patients were. The odds of being depressed decreased 17% per 10 nmol/L increase of calcidiol, which is significant. Symptom severity in dysthymic patients correlated with calcidiol but not in other groups. No significant differences were found in mean calcidiol levels between the four affective disorder diagnoses groups.
Calderon-Espinoza et al., 2022 [91]	Mexico; 72 patients were classified into three groups according to their vitamin D levels; 50.6 ± 12.76 years old	Determine the frequency of depression, anxiety symptoms, and suicidal risk or ideation, and associate it with vitamin D serum levels in patients with rheumatoid arthritis	Vitamin D quantification was determined using the chemiluminescence immunoassay technique (Liaison 25-OH Vitamin D Total Assay, Stillwater, MN)	Hospital Anxiety and Depression Scale (HADS)	Spanish adaptation of the self-applied Plutchik scale	Simplified Disease Activity Index (SDAI), Clinical Disease Activity Index (CDAI), Rheumatoid Arthritis Quality-of-Life Questionnaire, e-Health Assessment Questionnaire–Disability Index (HAQ–DI)	The Plutchik score and suicidal risk were inversely correlated with inadequate vitamin D levels but not with the Hospital Anxiety and Depression Scale. Higher scores on the Rheumatoid Arthritis Quality-of-Life Questionnaire were associated with suicidal ideation. Inadequate vitamin D serum levels correlated with a Plutchik low correlation coefficient. Regarding the covariance analysis, vitamin D levels persist associated with decreasing suicide ideation.

LC–MS, liquid chromatography–mass spectrometry; MDD, major depressive disorder; SI, suicidal ideation; DSM-IV-TR, Diagnostic and Statistical Manual of Mental Disorders, fourth edition, text revision; HDRS, hamilton depression rating scale; SCID, structured clinical interview for DSM-IV-TR axis I disorders; CPRS, comprehensive psychopathological rating scale; MADRS, Montgomery-Åsberg depression rating scale; SUAS-S, assessment scale, HADS, hospital anxiety and depression scale; SDAI, simplified disease activity index; CDAI, clinical disease activity index; HAQ–DI, health assessment questionnaire disability index.

### 5.3. Vitamin D and Neurological Mechanisms Associated with Depression and Suicide

Vitamin D is a key regulator of brain serotonin synthesis through *TPH2* gene expression, which contains a VDRE consistent with activation; therefore, low vitamin levels could be associated with low serotonin levels and psychiatric and mood disorders [62,98].

On the other hand, autopsies have shown elevated *VDR* mRNA expression in the brains of depressive individuals who died by suicide [99]. In this sense, some authors proposed that the VDREs would respond to vitamin D hormone in an inverse mode, with *TPH2* being transcriptionally activated in the brain and *TPH1* repressed in tissues outside of the blood-brain barrier (BBB) [100]. This proposal is based on evidence that the VDRE sequence alone can determine whether vitamin D will activate or repress gene transcription [101] and in a previous report showing that vitamin D activates *TPH2* expression in cultured neuronal cells [86].

Vitamin D has an important role in maintaining the vitality of neurons, such as those secreting neurotransmitters. After detecting the presence of VDR in the hippocampus, it was revealed that vitamin D is a potent modulator of the expression of the nerve growth factor (NGF), brain-derived neurotrophic factor (BDNF), and neurotrophin (NT)-3, which are necessary for the viability, growth, and migration of neurons. Therefore, it has been proposed that sufficient vitamin D levels could be associated with homeostatic neurotransmitter levels and with a minor risk of mood disorders such as depression [23,102].

Additionally, experimental models of depression have found that vitamin D contributes to improved serotonergic metabolism in the brain, as it not only increases serotonin synthesis by induction of the *TPH2* gene expression but influences the expression of serotonin reuptake transporter (SERT) and the levels of monoamine oxidase-A (MAO-A), responsible to serotonin catabolism. Therefore, the deregulation of vitamin D can alter these processes and favors depressive symptoms [103].

**Table 2:** Compilation of studies investigating the relationship between vitamin D and depression.

Author, Year	Country; *N*; Age	Objective	Vitamin D Measure	Depression Measure	Intervention	Additional Outcomes	Results/Conclusion
Lansdowne et al., 1998 [95]	Australia; 44 healthy students; 18 to 43 years old	Test the efficiency of vitamin D supplementation on participants’ mood during winter	No measurement	The Positive and Negative Affect Schedule (PANAS) was used as a self-report measure of positive affectivity (PA) and negative affectivity (NA)	Subjects were given 400 IU, 800 IU, or no vitamin D3 for five days during late winter in a random double-blind study	None	Both doses (400 IU and 800 IU) increased their reported PA by almost a full standard deviation above their population mean. NA did not decrease significantly for either dose group compared to the placebo. Even though it did not reach significance, the trend for NA was a decrease in both dose groups. The placebo group remained practically matched the population means.
Hoogendijk et al., 2008 [94]	The Netherlands; 1282 residents; 65 to 95 years old	Explore if there is an association between altered calcidiol and parathyroid hormone (PTH) levels and depression	Serum calcidiol concentration was determined using a competitive binding protein assay (Nichols Institute Diagnostics Inc, San Juan Capistrano, California)	Depression was measured using self-reports (Centre for Epidemiologic Studies–Depression scale) and diagnostic interviews (Diagnostic Interview Schedule)	None	Potentially confounding factors and explanatory factors were also measured	Compared to 1087 control individuals, calcidiol levels were 14% lower in 169 persons with minor depression, whereas their PTH was 5% higher. Twenty-six persons with MDD also had a lower calcidiol level by 14% and higher PTH by 33%. Decreased serum calcidiol levels and increased serum PTH levels were significantly associated with depression severity (Center for Epidemiologic Studies Depression Scale).
Jorde et al., 2008 [96]	Norway; 441 subjects (BMI 28–47 kg/m); 21–70 years old		Serum calcidiol was determined by immunometric (electrochemiluminescence) using an automated clinical chemistry analyzer (Modular E170; Roche Diagnostics^®^)	Depressed mood was judged with the Beck Depression Inventory (BDI) at inclusion and the end of the study	20,000 or 40,000 IU vitamin D per week or placebo for one year in a random double-blind study	Blood samples were drawn for analysis of serum calcium, creatinine, and parathyroid hormone (PTH)	Patients with < 40 nmol L(-1) calcidiol levels had significantly more depressive traits as measured by the total and subscales of the BDI than patients with serum levels ≥ 40 nmol L(-1) calcidiol levels. The BDI scores improved significantly after one year in both groups with vitamin D supplementation but not in the placebo group.
Kaviani et al., 2020 [23]	Iran; 56 subjects with mild to moderate depression and no other psychiatric disorder; 18–60 years old	Assess the effects of vitamin D supplementation on consequent serum calcidiol, depression severity, and serotonin and oxytocin in patients with mild to moderate depression	The enzyme immunoassay (EIA) method was employed for assessing serum calcidiol (Euroimmun EIA kit, Lubeck, Germany)	Structural clinical diagnostic interview based on the DSM–IV criteria and Beck Depression Inventory-II (BDI-II) score	50,000 IU cholecalciferol/2 weeks and control (placebo) in an 8-week double-blind, randomized clinical trial	Intact parathormone (iPTH), serum oxytocin, and platelet serotonin	After eight weeks, significant changes in the calcidiol concentrations and BDI-II scores were observed in the intervention group compared to the controls. Differences between groups were not significant for oxytocin and serotonin, but oxytocin concentrations were significantly reduced in controls, and platelet serotonin increased more in controls.
Rhee et al., 2020 [104]	Republic of Korea; 1736 subjects; 19 to 76 years old	Explore the association between specific domains of depressive symptoms and serum calcidiol concentrations by each sex	25-Hydroxyvitamin D 125I RIA Kit (DiaSorin, Stillwater, MN, USA) using a 1470 WIZARD Gamma Counter (PerkinElmer, Turku, Finland)	Patient Health Questionnaire-9 (PHQ-9)	None	Other covariates such as sociodemographic information, lifestyle behaviors, and health factors	Log-transformed serum calcidiol concentrations and total PHQ-9 scores were associated significantly only in men after adjusting for various covariates. Moreover, the association between the cognitive and affective subscales and the serum calcidiol concentrations was significant only in men. No association was found in the somatic subscale.

MDD, major depressive disorder; PANAS, positive and negative affect schedule; PA, positive affectivity; NA, negative affectivity; PTH, parathyroid hormone; iPTH, intact parathormone; EIA, enzyme immunoassay; BDI-II, beck depression inventory-II; PHQ-9, patient health questionnaire-9.

### 5.4. Vitamin D and Inflammatory Mechanisms Associated with Depression and Suicide

Some studies show the potential role of vitamin D as a mediator in the link between inflammatory markers, depression, and suicide. Blood levels of calcidiol are lower in suicide attempters compared to non-suicidal depressed patients and healthy controls; additionally, vitamin D levels correlated negatively with IL-1β for all subjects and with IL-6 in non-suicidal depressed patients [105]. In addition, abnormal CRP levels (>10 mg/L) also had a significant association with depressive symptoms [88].

Although classic inflammatory markers such as CRP, IL-6, and TNF-α are associated with depression and vitamin D deficiency, they do not seem to be all the mediators of this link [24,106,107]. White blood cell count (WBC) and neutrophile-to-lymphocyte ratio (NLR) have also been proposed as possible mediators of this relationship [24,107,108].

Calcitriol inhibits the activation and signaling of nuclear factor κB (NF-κB), which regulates the expression of several genes involved in inflammatory and immune responses [109]. Therefore, treatment with calcitriol reduces proinflammatory cytokine expression [110] and inhibits T-cell proliferation [111].

**Figure 2:**
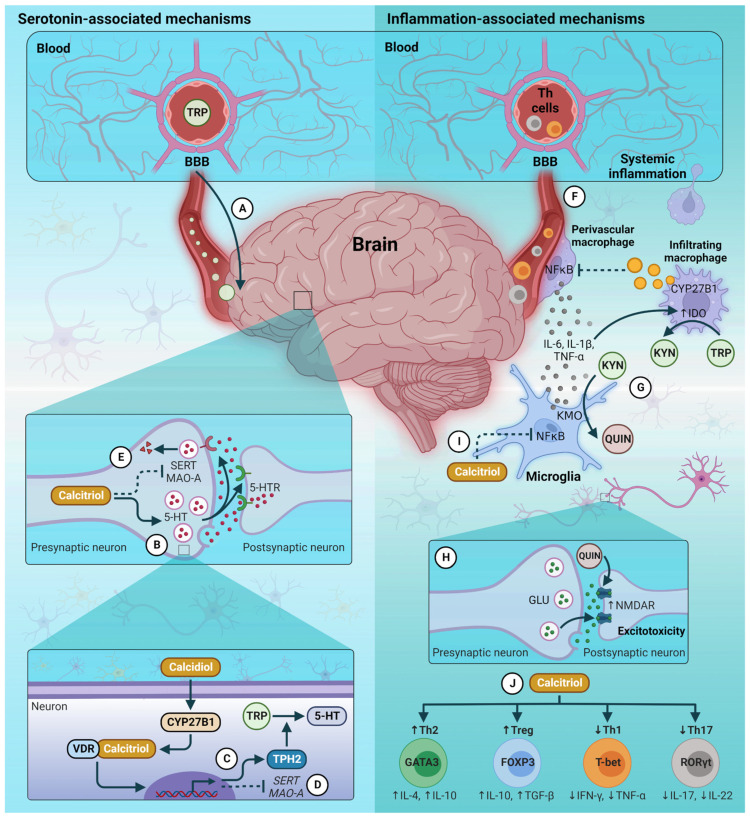
Protective mechanisms of vitamin D against depression. (**A**) TRP is an essential amino acid obtained from the diet. Dietary TRP that is not metabolized (e.g., in the intestine or liver) enters the circulation and can cross the BBB. (**B**) TRP is the primary amino acid precursor of 5-HT. This neurotransmitter is packaged in vesicles found in axon terminals. (**C**) Calcitriol produced by CNS cells has autocrine and paracrine effects. Calcitriol promotes the expression of TPH2, which is critical in the metabolism of TRP to 5-HT. (**D**) Likewise, calcitriol regulates *SERT* and *MAO-A* gene expression. (**E**) Therefore, calcitriol could decrease serotonin reuptake and its degradation, respectively. (**F**) The systemic inflammatory state due to pre-existing diseases promotes the infiltration of immune system cells into the brain. The production of proinflammatory cytokines (e.g., IL-6, IL-1β, and TNF-α) activates inflammatory signaling pathways that feed back into neuroinflammation. (**G**) Proinflammatory cytokines overregulate IDO activity so that TRP metabolism is redirected to the KYN pathway. Subsequently, KYN is converted to QUIN by the action of KMO in microglia. (**H**) QUIN increases NMDAR activity which triggers excessive GLU signaling leading to excitotoxicity. GLU excitotoxicity has effects on the pathogenesis of depression. (**I**) Calcitriol produced by macrophages, lymphocytes, and CNS cells has anti-inflammatory effects by suppressing NF-κB activity. (**J**) Calcitriol also has immunomodulatory effects that promote the production of anti-inflammatory cytokines by T cells. TRP, tryptophan; BBB, blood-brain barrier; 5-HT, serotonin; CNS, central nervous system; TPH2, tryptophan hydroxylase 2; CYP27B1, cytochrome P450 Family 27 Subfamily B Member 1 (25-hydroxyvitamin D-1 alpha hydroxylase; VDR, vitamin D receptor; SERT, serotonin reuptake transporter; MAO-A, monoamine oxidase-A; 5-HTR, serotonin receptor; IDO, indoleamine 2,3-dioxygenase; KYN, kynurenine; QUIN, quinolinic acid; KMO, kynurenine 3-monooxygenase; NMDAR, N-methyl-D-aspartic acid receptor; GLU, glutamate; NF-κB, nuclear factor κB; Th, T helper cells; Tregs, T regulatory cells.

The possible protective mechanisms of vitamin D against depression are summarized in Figure 2. Moreover, conclusions of previous studies on vitamin D, inflammation, and depression are shown in Table 3.

## 6. Relationship between *VDR* Gene, Depression, and Suicide

Polymorphisms in the *VDR* gene can alter its expression in several cells and tissues, including the brain [19,112,113,114]. Moreover, polymorphisms in this gene can alter the VDR’s function and reduce or enhance the expression of other genes induced by vitamin D [115].

A study compared the mRNA expression of the *VDR* gene in the dorsolateral prefrontal cortex (dlPFC) and anterior cingulate cortex (ACC) between depressed individuals who died by suicide and non-psychiatric controls. The results showed higher *VDR* expression in both dlPFC and ACC in suicides relative to controls [99]. Another study also reported an upregulation of the *VDR* gene in bipolar disorder, and it correlated with an elevated risk of premature death from suicide and comorbid conditions [116]. These changes in the *VDR* gene expression could be associated to genetic variants or epigenetic factors.

Variants of the *VDR* gene have previously been associated with susceptibility to depressive symptoms [117,118]. However, there are more than 200 polymorphisms reported in the *VDR* gene, several of them associated with biological effects and with inflammatory diseases. As expression and nuclear activation of the VDR are necessary for the effects of vitamin D. It would be valuable to analyze in the future the possible association of all these polymorphisms with depression and suicide [119].

## 7. Vitamin D Supplementation for the Treatment of Depression

Several randomized clinical trials (RCTs) have tested the effects of vitamin D supplementation on the treatment of depression. In a study conducted on Iranian patients diagnosed with irritable bowel syndrome and with baseline calcidiol concentrations below 30 ng/mL (75 nmol/L), supplementation with 50,000 IU (1250 μg) of vitamin D_3_ once weekly for nine weeks increased calcidiol concentration significantly (18.59 ± 7.58 vs. 46.86 ± 12 ng/mL, *p* < 0.001). In addition, after supplementation, the intervention group presented an improvement in the Hospital Anxiety and Depression Scale based on the decrease in the mean score (6.59 ± 4.63 vs. 5.26 ± 4.68, *p* = 0.008); however, the final score did not differ from the placebo group (5.26 ± 4.68 vs. 6.06 ± 3.8, *p* = 0.425) [22].

Likewise, another study in Iranian patients with mild to moderate depression implemented supplementation with 50,000 IU (1250 μg) of vitamin D_3_ every two weeks for eight weeks. The calcidiol concentration of the intervention group increased significantly (34.84 ± 11.42 vs. 51.17 ± 9.97 ng/mL, *p* < 0.001). Similarly, patients under the supplemental regimen presented an improvement in their depression score (Beck Depression Inventory-II) after the eight weeks of intervention (23.86 ± 5.49 vs. 12.11 ± 6.12, *p* < 0.001). The final score was significantly different from the placebo group’s (12.11 ± 6.12 vs. 18.18 ± 12.82, *p* = 0.003) [23].

A study in women with type 2 diabetes identified that vitamin D_3_ supplementation for six months effectively improves depressive symptoms regardless of the dose of vitamin D_3_, either 5000 IU (125 μg) per day or 50,000 IU (1250 μg) once a week. However, the increase in serum calcidiol was more noticeable in the high-dose vitamin D_3_ group after six months of supplementation (30.29 vs. 55.55 ng/mL) [120]. Likewise, other research groups have reported positive effects of vitamin D administration on depressive symptoms with different doses and routes of administration (4000 IU per day during 3 months or a single parenteral dose of 300,000 IU) [121,122]. On the contrary, several studies have reported no significant effects of vitamin D on depression [123,124,125,126].

According to a recent meta-analysis, the primary outcome involving 41 RCTs demonstrated that vitamin D supplementation has minor to moderate effects on depressive symptoms (Hedges’ *g* = −0.317 (95% CI: −0.405 to −0.230), *p* < 0.001, *I*^2^ = 88.16%). Although vitamin D supplementation may have effects in patients with mild depressive symptoms, the effect was more significant in patients with clinically relevant depressive symptoms (Hedges’ *g* = −0.604 (95% CI: −0.802 to −0.406), *p* < 0.001, *I*^2^ = 78.4%). Moreover, the meta-analysis identified that the effect was greater in those studies in which a dose over 2000 IU (50 μg) of vitamin D was administered (Hedges’ *g* = −0.407 (95% CI: −0.556 to −0.259), *p* < 0.001, *I*^2^ = 75.8%). Nevertheless, the present results showed high heterogeneity and significant evidence for potential publication bias [127]. Despite the inconsistent results, maintaining a sufficient calcidiol concentration is considered beneficial for maintaining an overall good health status [11,13,29].

## 8. Conclusions

The present review can be seen as consistent with the potential pathogenic role of vitamin D deficiency in depression and suicide. Several studies have shown that deficiencies in this vitamin reduce the immunomodulation of inflammation and serotonin synthesis, two processes associated with depression and suicidal attempts. Therefore, it supports the potential benefits of vitamin D supplementation in reducing symptoms of depression and a possible indirect effect in the prevention of suicide and suicide attempts.

Vitamin D levels determination and supplementation with vitamin D are affordable and safe. Thus, both actions could be good routine clinical processes in patients with suicidal symptoms. However, more clinical trials are required to determine with greater precision the best way to supplement or obtain vitamin D, including duration, doses, or routines of sun exposure. Moreover, it is pertinent that in vitro studies clarify the role of VDR in the brain and the possible increase in its expression in case of depression or suicide. This will make it possible to propose better analysis strategies for the link between vitamin D and these clinical entities.

## Figures and Tables

**Table 3:** Studies on the relationship between vitamin D, inflammation, and depressive symptoms.

Author, Year	Participants	Conclusions
Shin et al., 2016 [88]	52,228	No direct association between serum vitamin D and serum CRP levels; Increased OR for depressive symptoms in patients with vitamin D insufficiency (10–19.99 ng/mL) and deficiency (<10 ng/mL); Positive association and increased OR for depressive symptoms and abnormal serum (>10 mg/L) CRP levels.
Dogan-Sander et al., 2021 [107]	7162	Correlation between the Center for Epidemiologic Studies Depression Scale (CES-D) with calcidiol and inflammatory markers; WBC is a possible mediator of calcidiol and CES-D relationship. Inflammatory markers do not act as mediators.
Nerhus et al., 2016 [108]	358	Low calcidiol associated with depression; CRP correlates with calcidiol and negative symptoms, no mediation effect.
Grudet et al., 2020 [24]	102	Higher correlation between calcidiol and inflammatory markers in depressed patients with suicidal ideation; Major depressive disorder moderated the relationship between calcidiol with NLR and WBC.
Grudet et al., 2014 [90]	90	Lower vitamin D levels in suicide attempters compared to those non-suicidal and healthy controls; Low vitamin D levels are associated with higher levels of IL-6 and IL-1β.
Hashash et al., 2019 [106]	1352	Inflammatory bowel disease patients with suicidal ideation had lower levels of vitamin D.
Calderón-Espinoza et al., 2022 [91]	72	Correlation between CES-D with calcidiol and inflammatory markers.

CRP, C-reactive protein; OR, odds ratio; CES-D, Center for Epidemiologic Studies Depression Scale; WBC, white blood cell count; NLR, neutrophile-to-lymphocyte ratio.

## Data Availability

No new data were created or analyzed in this study. Data sharing is not applicable to this article.

## Abbreviations

1,25(OH)_2_D	calcitriol
25(OH)D	calcidiol
7-DHC	7-dehydrocholesterol
ACC	anterior cingulate cortex
BBB	blood-brain barrier
BDNF	brain-derived neurotrophic factor
CRP	C-reactive protein
CYP27B1	25-hydroxyvitamin D-1 alpha hydroxylase
CYP2R1	25-hydroxylase
DBP	vitamin D binding protein
dlPFC	dorsolateral prefrontal cortex
HPA	hypothalamic–pituitary–adrenal
IDO	indoleamine 2,3-dioxygenase
IL	interleukin
KYN	kynurenine
LPS	lipopolysaccharide
MAO-A	monoamine oxidase-A
MDD	major depressive disorder
NF-κB	nuclear factor κB
NGF	nerve growth factor
NLR	neutrophile-to-lymphocyte ratio
NT	neurotrophin
QUIN	quinolinic acid
RCT	randomized clinical trials
RXR	retinoid X receptor
SAD	seasonal affective disorder
SERT	serotonin reuptake transporter
SSRI	selective serotonin reuptake inhibitors
TPH2	tryptophan hydroxylase 2
UVB	ultraviolet-B
VDR	vitamin D receptor
VDREs	vitamin D response elements
WBC	white blood cell count
WHO	World Health Organization
